# Supplementary material for: Empirical methods for controlling false positives and estimating confidence in ChIP-Seq peaks
Source: BMC Bioinformatics. 2008 Dec 5;9:523. doi: 10.1186/1471-2105-9-523 (PMC2628906; doi:10.1186/1471-2105-9-523)
Supplement: Additional File 1 — A variety of html documents from the USeq web site detailing the available applications, their best usage, output file type descriptions, command line menus, etc. [file 1471-2105-9-523-S1.zip › USeqUserGuides/JavaDoc.zip!/JavaDoc/bioroot/strain/class-use/StrainBean.html]

Uses of Class bioroot.strain.StrainBean


|  |  |  |
| --- | --- | --- |
| **Overview** \| **Package** \| **Class** \| **Use** \| **Tree** \| **Deprecated** \| **Index** \| **Help** | |  |
| PREV   NEXT | **FRAMES**    **NO FRAMES**     **All Classes** |  |


---


## **Uses of Class bioroot.strain.StrainBean**

| Packages that use StrainBean | |
| --- | --- |
| **bioroot** |  |
| **bioroot.strain** |  |

Uses of StrainBean in bioroot

| Methods in bioroot that return StrainBean | |
| --- | --- |
| `static StrainBean[]` | `Archive.makeStrainBeans(DBUtil bioRoot, UserBean userBean)`             Makes an array of all visibile StrainBeans |

| Methods in bioroot with parameters of type StrainBean | |
| --- | --- |
| `static java.util.ArrayList` | `Archive.fetchFiles(PlasmidBean[] plasmids, StrainBean[] strains, AntibodyBean[] antibodies, java.lang.String filePath, DBUtil bioRoot)`             Fetches any plasmid or strain files. |

Uses of StrainBean in bioroot.strain

| Methods in bioroot.strain that return StrainBean | |
| --- | --- |
| `static StrainBean[]` | `StrainSpreadSheet.fetchStrainBeans(java.lang.String sql, DBUtil bioRoot)`             Build StrainBean[] from Strain table in database. |
| `StrainBean[]` | `StrainUploadBean.getStrainBeans()` |
| `static StrainBean[]` | `StrainSpreadSheet.orderStrains(java.lang.String featureName, UserBean userBean, StrainBean[] beans, DBUtil bioRoot)`             Orders strains, sort of nasty, can't figure another way, reflection is way too slow. |
| `static StrainBean[]` | `StrainBase.reverseStrainBeans(StrainBean[] beans)`             Reverses the order of an array of StrainBeans. |

| Methods in bioroot.strain with parameters of type StrainBean | |
| --- | --- |
| `static void` | `NewStrain.addParent(StrainBean bean, java.util.HashMap nameValue, DBUtil bioRoot, UserBean userBean)` |
| `static void` | `NewStrain.addPlasmid(StrainBean bean, java.util.HashMap nameValue, DBUtil bioRoot, UserBean userBean)` |
| `static void` | `StrainUpload.appendStrainBeans(javax.servlet.http.HttpSession session, StrainBean[] newBeans)`             Sets strainBeans attribute by appending any new beans to the beginning. |
| `static java.lang.String` | `StrainModify.deleteStrain(StrainBean strainBean, UserBean userBean, DBUtil bioRoot)`             Attempts to delete an strain, if sucessful, returns a "" String, otherwise an error message. |
| `static java.lang.String` | `StrainSpreadSheet.fetchStrainTableRows(UserBean userBean, StrainBean[] beans, int[] startStop, DBUtil bioRoot, boolean checked, boolean allTabs)`             Returns table rows for every oligo according to a Preference, if allTabs==true, then all features are returned in a tab delimited format, otherwise its HTML. |
| `static java.lang.String` | `StrainBase.getStrainForm(StrainBean sb, DBUtil bioRoot, UserBean userBean)`             Returns a strain form filled with the values in the bean. |
| `static java.lang.String` | `StrainBase.getStrainReport(StrainBean sb, DBUtil bioRoot, UserBean userBean, boolean commonForm)`             Returns an strain report. |
| `static StrainBean[]` | `StrainSpreadSheet.orderStrains(java.lang.String featureName, UserBean userBean, StrainBean[] beans, DBUtil bioRoot)`             Orders strains, sort of nasty, can't figure another way, reflection is way too slow. |
| `static void` | `NewStrain.removeParent(StrainBean bean, java.util.HashMap nameValue, DBUtil bioRoot)` |
| `static void` | `NewStrain.removePlasmid(StrainBean bean, java.util.HashMap nameValue, DBUtil bioRoot)` |
| `static StrainBean[]` | `StrainBase.reverseStrainBeans(StrainBean[] beans)`             Reverses the order of an array of StrainBeans. |
| `void` | `StrainUploadBean.setStrainBeans(StrainBean[] beans)` |
| `static boolean` | `StrainModify.subtractAndSetStrainBeans(StrainBean[] strainBeans, javax.servlet.http.HttpSession session)`             Subtracts a bean from the StrainBean[] and resets it in strainBeansToEdit |
| `static java.lang.String` | `StrainModify.updateStrain(StrainBean bean, UserBean userBean, java.util.HashMap nameValue, javax.servlet.http.HttpServletRequest request, DBUtil bioRoot)`             Attempts to update a StrainBean in the database, only checks that the name is unique if it has changed. |
| `static boolean` | `StrainModify.updateStrainCommonAccessFields(StrainBean bean, UserBean userBean, java.util.HashMap nameValue, DBUtil bioRoot)`             Just update the commonly accessible fields: restock, lastUser, remainingAliquots, comments |

---


|  |  |  |
| --- | --- | --- |
| **Overview** \| **Package** \| **Class** \| **Use** \| **Tree** \| **Deprecated** \| **Index** \| **Help** | |  |
| PREV   NEXT | **FRAMES**    **NO FRAMES**     **All Classes** |  |


---
